# Death-Associated Protein 6 (Daxx) Alleviates Liver Fibrosis by Modulating Smad2 Acetylation

**DOI:** 10.3390/cells10071742

**Published:** 2021-07-09

**Authors:** Sung-Min Kim, Won-Hee Hur, Byung-Yoon Kang, Sung-Won Lee, Pu-Reun Roh, Dong-Jun Park, Pil-Soo Sung, Seung-Kew Yoon

**Affiliations:** 1 The Catholic University Liver Research Centre, Department of Biomedicine & Health Sciences, POSTECH-Catholic Biomedical Engineering Institute, College of Medicine, The Catholic University of Korea, Seoul 06591, Korea; wildtultul@catholic.ac.kr (S.-M.K.); wendyhur@korea.kr (W.-H.H.); kby2132@catholic.ac.kr (B.-Y.K.); zambrotta@catholic.ac.kr (S.-W.L.); shvn4564@catholic.ac.kr (P.-R.R.); akhpdyj@catholic.ac.kr (D.-J.P.); pssung@catholic.ac.kr (P.-S.S.); 2 Division of Hepatology, Department of Internal Medicine, Seoul St. Mary’s Hospital, College of Medicine, The Catholic University of Korea, 222, Banpo-daero, Seocho-gu, Seoul 06591, Korea

**Keywords:** liver fibrosis, epithelial–mesenchymal transition, death-associated protein 6, transforming growth factor-β, Smad2

## Abstract

Transforming growth factor-β (TGF-β) has been identified as an inducer of hepatocyte epithelial–mesenchymal transition (EMT), which triggers liver fibrosis. Death-associated protein 6 (Daxx) is known to be associated with the TGF-β-induced apoptotic pathway, but the function of Daxx in liver fibrosis remains unknown. This study aimed to elucidate the role of Daxx in liver fibrosis. We used liver fibrosis tissues from humans and mice to assess Daxx expression. EMT properties and TGF-β signaling pathway activation were investigated in the Daxx-overexpressing FL83B cell line. The therapeutic effect of Daxx was investigated in a mouse model of liver fibrosis by the hydrodynamic injection of plasmids. The expression of Daxx was markedly decreased in hepatocytes from fibrotic human and mouse livers, as well as in hepatocytes treated with TGF-β in vitro. The overexpression of Daxx inhibited the EMT process by interfering with the TGF-β-induced phosphorylation of Smad2. Coimmunoprecipitation analysis confirmed that Daxx reduced the transcriptional activity of Smad2 by binding to its MH1 domain and interfering with Smad2 acetylation. In addition, the therapeutic delivery of Daxx alleviated liver fibrosis in a thioacetamide-induced fibrosis mouse model. Overall, our results indicate that Daxx could be a potential therapeutic target to modulate fibrogenesis, as well as a useful biomarker for liver fibrosis.

## 1. Introduction

Liver fibrosis is a pathological consequence of repeated inflammation and repair mechanisms resulting from various causes, such as viral infections, obesity, alcohol consumption, or drugs. Moreover, liver fibrosis can lead to cirrhosis, which is one of the leading causes of morbidity and mortality worldwide. The burden of liver cirrhosis, which causes portal hypertension, hepatocellular carcinoma and eventual hepatic failure, has consistently increased over the past thirty years, rising from 676,000 related deaths in 1980 to over one million related deaths in 2010; despite this rise in cases, an antifibrotic agent for the treatment of liver fibrosis has not yet been developed [1,2].

Liver fibrosis is characterized by an excessive accumulation of extracellular matrix (ECM) proteins, including collagen, which deform the hepatic architecture by forming a fibrous scar. Myofibroblasts are the major ECM-producing cells and are normally derived from hepatic stellate cells (HSCs) in the liver [3]. It has been reported that damaged hepatocytes can also be converted into myofibroblast-like cells via epithelial–mesenchymal transition (EMT) [4,5]. EMT is a dynamic cellular process by which polarized immotile epithelial cells lose their typical epithelial properties due to a reduction in the expression of epithelial signature genes, such as E-cadherin, Zo-1 and cytokeratin and become motile mesenchymal-like cells through the upregulation of the expression of mesenchymal signature genes, such as Vimentin, N-cadherin and fibronectin. EMT has been observed in physiological processes, such as development and wound healing, but it is also associated with pathological processes, such as fibrotic diseases and cancer metastasis [6,7,8].

Activation of HSCs and induction of hepatocyte EMT are induced by the profibrogenic cytokine transforming growth factor-beta (TGF-β) [9]. TGF-β is a signaling molecule involved in multiple cellular processes and it plays an important role in regulating cellular development processes, such as growth, proliferation and apoptosis. The TGF-β signaling pathway begins when TGF-β binds to the serine/threonine kinase TGF-β receptors and induces the phosphorylation of Smad2/3, which subsequently oligomerizes with Smad4. The Smad2/3 and Smad4 complex translocates into the nucleus, where it initiates the transcription of fibrotic target genes. Therefore, due to the major role of TGF-β in fibrogenesis, the TGF-β pathway has become one of the key targets for the development of effective antifibrotic therapies [10,11,12]. However, TGF-β signaling-based antifibrotic therapies have not yet been successful due to the complexity of this signaling pathway and cell type-dependent outcomes [13]. Therefore, a better understanding is required in relation to the precise molecular mechanism that regulates the TGF-β signaling pathway.

Death domain-associated protein (Daxx) was initially identified as a novel Fas-binding protein that enhances FAS-mediated cellular apoptosis [14]; it is also known to be associated with TGF-β-induced apoptosis through JNK activation [15]. Daxx is known to be present in the cytoplasm and nucleus and, when Daxx modulates cell death in the cytoplasm, it interacts with various DNA-binding transcription factors (TFs) within the nucleus, acting as a transcriptional repressor of p53 and NF-κB [16,17]. Recent studies have revealed that Daxx has the ability to regulate cellular processes by modulating the transcription of certain genes in various types of disease [18,19]. However, the role of Daxx in liver disease, especially liver fibrosis, has yet to be studied. Therefore, we aimed to elucidate the role of Daxx in liver fibrosis and sought to outline the precise underlying mechanisms associated with the TGF-β signaling pathway.

## 2. Materials and Methods

### 2.1. Animal Studies

Six-week-old C57BL/6 (Orient Bio Inc, Seoul, Korea) mice were administered thioacetamide (TAA, Sigma-Aldrich, St Louis, MO, USA) to induce liver fibrosis. The mice were randomly divided into three groups: the normal group, the TAA-injected group (TAA, 100 mg/kg body weight) and the TAA with HA-Daxx plasmid injection group (TAA + Daxx, 30 mg/mouse via a hydrodynamic injection system). TAA injections were given every 3 days for 10 weeks and Daxx hydrodynamic injections were given every 3 days for 5 weeks. All the procedures were conducted in accordance with the Laboratory Animals Welfare Act, the Guide for the Care and Use of Laboratory Animals and the Guidelines and Policies for Rodent Experiments provided by the Institutional Animal Care and Use Committee (IACUC) of the Catholic University of Korea (approval number: CUMC-2015-0145-02, 31 August 2015).

### 2.2. Cell Culture

Normal mouse hepatocyte cell line FL83B (ATCC CRL-2390) was cultured in Ham’s F-12 K medium containing 10% fetal bovine serum (FBS), 100 mg/mL penicillin and 0.25 mg/mL streptomycin and HEK 293T cells (ATCC CRL-3216) were cultured in Dulbecco’s modified Eagle’s medium (DMEM), containing 10% FBS, 100 μg/mL penicillin and 0.25 μg/mL streptomycin (all from Invitrogen, Carlsbad, CA, USA). Both cell lines were purchased from the American Type Culture Collection (Rockville, MD, USA). The cells were maintained at 37 °C in a humidified incubator with 5% CO_2_. To induce EMT, FL83B cells were seeded on 100 mm plates (2 × 10^5^ cells). Twenty-four hours after seeding, the cells were treated with 3 ng/mL of TGF-β1 (R&D Systems, Minneapolis, MN, USA) in 0.5% FBS-containing medium for 24 h. For the overexpression of Daxx, Smad2, MH1 and MH2, FL83B and HEK 293T cells were seeded on 100 mm plates (FL83B cells: 2 × 10^5^ cells; HEK 293T cells: 5 × 10^5^ cells). After 24 h, the cells were transfected with pcDNA3.0-HA-tagged Daxx expression vector (Science Reagents #1-0012-0004), pCS2+ FLAG-tagged Smad2, pCS2+ FLAG-tagged MH1, or pCS2 + FLAG-tagged MH2 (CS2 Flag-Smad2, Smad2 (1-185) and Smad2 (100-467) were a gift from Joan Massague (Addgene plasmid #14042, #14929, #14928)), using Fugene HD (Promega, Madison, WI, USA) for 24 h. Then, the cells were treated with 3 ng/mL of TGF-β1.

### 2.3. Coimmunoprecipitation and Cell Fractionation

HEK 293T cells were transfected with HA-tagged Daxx with Flag-tagged Smad2, MH1, or MH2 for 24 h and lysed in lysis buffer (50 mM Tris-Cl (pH 7.5), 120 mM NaCl, 0.5% (*v*/*v*) NP-40, 50 mM NaF, 200 mM sodium orthovanadate and 1 mM phenylmethylsulfonyl fluoride). The protein extracts (500 mg) were incubated with anti-FLAG beads (Sigma-Aldrich), anti-HA antibody (Santa Cruz Biotechnology, Santa Cruz, CA, USA), or anti-acetyl lysine antibody (Cell Signaling, Beverly, MA, USA) overnight and then precipitated with anti-HA and anti-acetyl lysine antibodies using protein A agarose (Invitrogen, Carlsbad, CA, USA). After 1 h, the beads were washed with ice-cold PBS and the bound immunoprecipitates were eluted from the beads by boiling in sample buffer (62.5 mM Tris–HCl (pH 6.8), 10% glycerol, 2% sodium dodecyl sulfate (SDS), 144 mM β-mercaptoethanol and 0.0005% bromophenol blue). For cell fractionation, the nuclear and cytoplasmic fractions were extracted from FL83B and HEK 293T cells using an NE-PER kit (Pierce Biotechnology, Rockford, IL, USA), according to the manufacturer’s protocol.

### 2.4. Western Blotting

The cells and liver tissues were lysed in RIPA buffer (20 mM Tris-HCl at pH 7.5, 150 mM NaCl, 1% Triton X-100, 1% sodium deoxycholate and 0.1% SDS) containing protease inhibitor cocktail (Roche, Basel, Switzerland) and phosphatase inhibitor cocktails (100×) (Sigma-Aldrich). SDS-PAGE (10%) was conducted to separate the protein extracts; then, the proteins were transferred to nitrocellulose membranes (Schleicher & Schuell, Dassel, Germany). After blocking the membrane in TBS containing 5% skim milk (10 mM Tris-HCl at pH 7.5 and 150 mM NaCl) for 30 min, the membrane was incubated overnight at 4 °C with primary antibodies (Supplementary Table S1). The specific protein bands were visualized with an enhanced chemiluminescent system (ECL, Amersham Pharmacia Biotech., Arlington Heights, IL, USA). The images were captured using an LAS-4000 (Fuji-Film, Tokyo, Japan) imaging system.

### 2.5. Quantitative Real-Time PCR

To detect the expression of mRNAs, complementary DNA (cDNA) was synthesized as previously described [20]. PCR was performed using specific primers. The primer sequences were as follows (5′→3′): Snal1 sense CCACACTGGTGAGAAGCCATTC and antisense TCTTCACATCCGAGTGGGTTTG; Slug sense TGTATGGACATCGTCGGCAG and antisense ACTTACACGCCCCAAGGATG; Zeb1 sense ACTGCCAGCAGACCAGAC A and antisense TCACACTCGTTGTCTTTCACG; Zeb2 sense TGCGTCCACTACGTT GTCAT and antisense CAAGAGGCGCAAACAAGC. TaqMan probe-based real-time PCR amplifications were performed with a Light Cycler 480 instrument (Roche Applied Science, Indianapolis, IN, USA) in a total reaction volume of 20 mL.

### 2.6. Immunohistochemistry (IHC) and Immunofluorescence (IF)

IHC and IF were performed as previously described [21]. For IHC, the purchased human paraffin embedded tissue array (TMA) (LV805a, US Biomax, Rockville, MD, USA) and mouse liver tissues were permeabilized with PBST (0.1% Triton X-100 in PBS), blocked with 5% goat serum and incubated with primary antibodies overnight. The antibodies used in immunohistochemistry were Daxx (1:25) and Smad2/3 -> Smad2 (1:50). Images were scanned using a Pannoramic MIDI slide scanner (3D HISTECH, Budapest, Hungary). To quantify the Daxx positive cells, IHC images were captured using a Pannoramic viewer (3D HISTECH). Hepatocyte nuclei of whole liver and Daxx-positive cells were counted using Optimas 6.5 software (Agris-Schoen Vision System, Alexan-dria, VA, USA). Then, Daxx-positive cells were calculated as Daxx-positive nuclei/Total nuclei × 100.

For IF, FL83B cells were fixed in 4% PFA and stained with F-actin (1:100), Daxx (1:25), HA (1:50) and Smad2/3 -> Smad2 (1:50) antibodies overnight. DAPI (Sigma-Aldrich) was used for counterstaining. The fluorescence was detected by an LSM800 w/Airyscan confocal microscope (Carl Zeiss, Oberkochen, Germany).

### 2.7. Measurement of Liver Collagen

Sirius red staining was performed using the Picro–Sirius Red Staining Kit (Abcam, Cambridge, UK) to evaluate collagen deposition in liver tissues. For collagen deposition determination, a series of 5 randomly selected fields from each slice were visualized and quantified using ImageJ 1.48v software (National Institutes of Health, Bethesda, MD, USA). To detect the level of hepatic hydroxyproline, a specific component of collagen, a hydroxyproline assay kit (BioVision, Milpitas, CA, USA) was used, according to the manufacturer’s protocol.

### 2.8. Statistical Analysis

All the results are expressed as the mean ± SEM. Comparisons between two groups were performed using two-tailed Student’s *t* test. * *p* < 0.05. ** *p*  <  0.01. *** *p*  <  0.001. All the experiments were performed at least three times. Statistical analysis was performed using GraphPad Prism version 7 (GraphPad software, San Diego, CA, USA).

## 3. Results

### 3.1. The Expression of Daxx Was Decreased in Fibrotic Human and Mouse Livers

**Figure 1:**
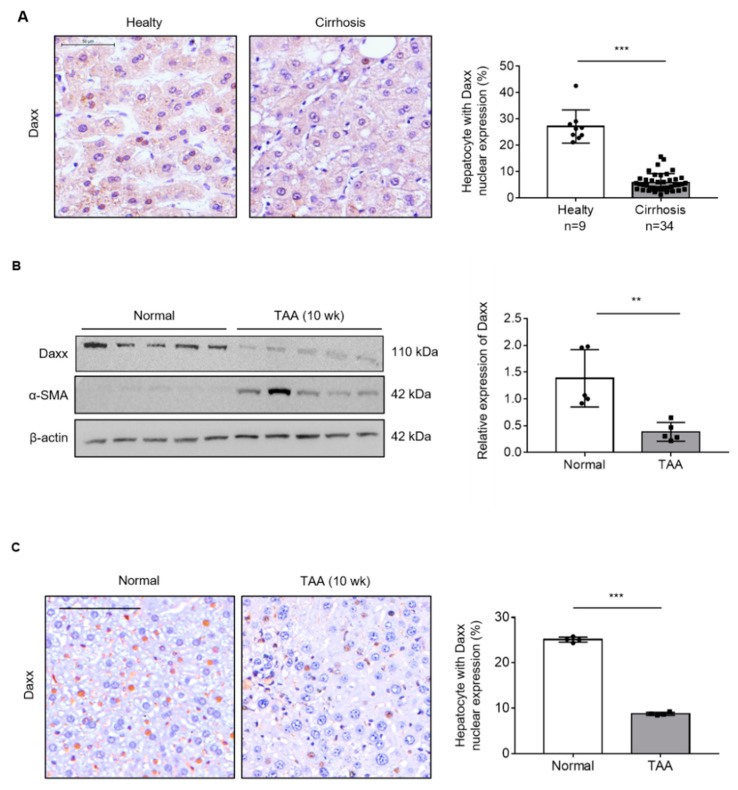
The expression of Daxx was decreased during liver fibrosis. (**A**) The protein expression of Daxx was detected by IHC in healthy or cirrhotic liver tissues from patients. The nuclear expression of Daxx was quantified by Optimas 6.5 software. Original magnification: 100×. (**B**) The protein levels of Daxx and α-SMA were determined in normal and TAA-induced fibrotic mouse liver tissues by Western blotting. The relative expression was normalized to β-actin expression as a reference. The graph shows quantitative densitometry from the Western blot. (**C**) Dissected fibrotic liver tissues were stained with Daxx. Original magnification: 100x. Nuclear expression of Daxx was counted by Optimas 6.5 software in 4 liver tissues per group. The data are the mean ± SEM. ** *p* < 0.01, *** *p* < 0.001.

To determine the role of Daxx in liver fibrosis, we assessed Daxx expression in human healthy (n = 9) and cirrhotic liver (n = 34) tissues using TMA. The IHC data showed that Daxx was most abundantly expressed in the nuclei of hepatocytes; thus, we counted Daxx-positive hepatocytes nuclei from total hepatocyte nuclei and calculated them as a percentage. The result of our experiment demonstrated that Daxx-positive cells were reduced in cirrhotic liver tissues compared to healthy tissues from human livers (Figure 1A). Then, we assessed the expression of Daxx in a TAA-induced fibrotic liver mouse model using Western blotting and IHC. Western blotting analysis showed that Daxx expression was dramatically decreased in the liver of TAA-induced fibrotic mice, with high expression of the fibrosis marker, α-smooth muscle actin (α-SMA) compared to normal mice liver. (Figure 1B). Furthermore, IHC staining of Daxx showed that Daxx was most expressed in the nuclei of normal mouse hepatocytes, as shown in Figure 1A, and that its expression was decreased in TAA-treated fibrotic mouse hepatocytes. Therefore, we counted and quantified the cells expressing Daxx in the nucleus. The quantification graph showed that the Daxx-positive cells were decreased by approximately 80% in fibrotic livers compared to normal livers (Figure 1C). These results clearly showed that Daxx expression was reduced in the nuclei of hepatocytes in fibrotic livers compared to normal livers and it can be expected that Daxx is associated with liver fibrosis.

### 3.2. Restoration of Daxx Expression Inhibits TGF-β-Induced Hepatocyte EMT

**Figure 2:**
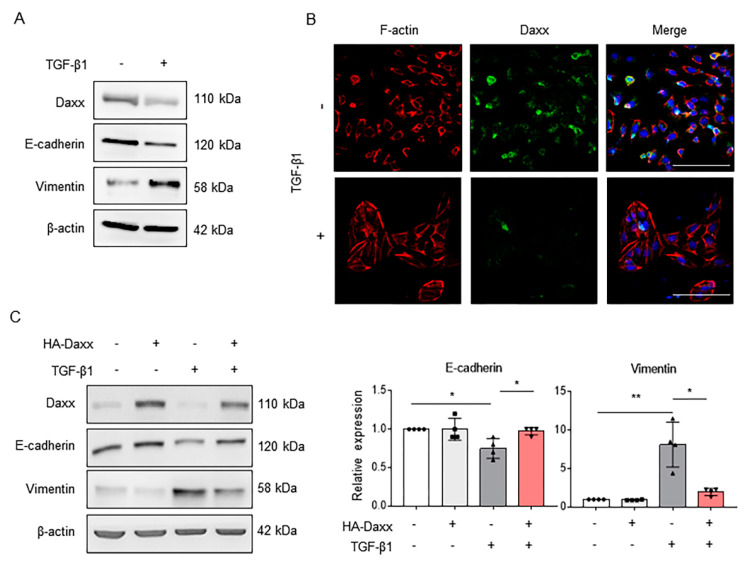
Daxx inhibited TGF-β-induced hepatocyte EMT. (**A**) Western blotting analysis of Daxx, E-cadherin and Vimentin expression in 3 ng/mL of TGF-β-treated or untreated FL83B cells. (**B**) Immunofluorescence staining of F-actin (red) and Daxx (green). DAPI was used to counterstain the nucleus. Original magnification: 100×. (**C**) FL83B cells were transfected with HA-Daxx and then treated with or without TGF-β for 24 h. Western blotting analysis of Daxx, E-cadherin and Vimentin expression. Relative expression was normalized to β-actin expression as a reference. The graph shows quantitative densitometry from Western blotting. The data are the mean ± SEM. The results are representative of at least three independent experiments. * *p* < 0.05, ** *p* < 0.01.

Our previous study showed that hepatocytes are associated with liver fibrosis via the EMT process [20]. As seen from the IHC data in Figure 1B, Daxx was also expected to be associated with EMT because of its expression in hepatocytes. Thus, we established a TGF-β1-induced EMT model in the hepatocyte cell line FL83B and then confirmed Daxx expression using Western blotting. After 24 h of TGF-β treatment, the expression of Daxx and an epithelial marker, E-cadherin, was markedly diminished and expression of the mesenchymal marker Vimentin was increased (Figure 2A). Then, we visualized the distribution of filamentous actin (F-actin) using rhodamine-phalloidin staining, because actin cytoskeletal reorganization is induced during the EMT process. The results showed that F-actin filaments (red color) in TGF-β-treated cells were changed from short fibers to long fibers, compared to those in untreated cells, and Daxx expression (green color) was decreased in TGF-β-treated cells (Figure 2B). These results confirmed that Daxx expression was downregulated in hepatocytes exhibiting EMT characteristics.

To investigate the functional impact of Daxx on the EMT process, we overexpressed Daxx with the HA-tagged Daxx plasmid and then treated the cells with TGF-β for 24 h. As shown in Figure 2A, the expression of E-cadherin was decreased but the expression of Vimentin was increased by TGF-β treatment. In contrast, in the presence of Daxx, the expression of E-cadherin and Vimentin remained unchanged both with and without TGF-β treatment (Figure 2C). On the other hand, as a result of measuring a PCNA, a proliferation marker, hepatocytes proliferation was not changed by Daxx overexpression (Figure S1A). Moreover, we measured mRNA levels of α-SMA and collagen type I (col1a1), important markers for ECM production and accumulation, which were increased after TGF-β treatment, but they were inhibited by Daxx overexpression (Figure S1B). These results suggest that TGF-β-induced hepatocyte EMT can be prevented by restoring Daxx expression.

### 3.3. Daxx Expression Inhibited the Phosphorylation of Smad2

**Figure 3:**
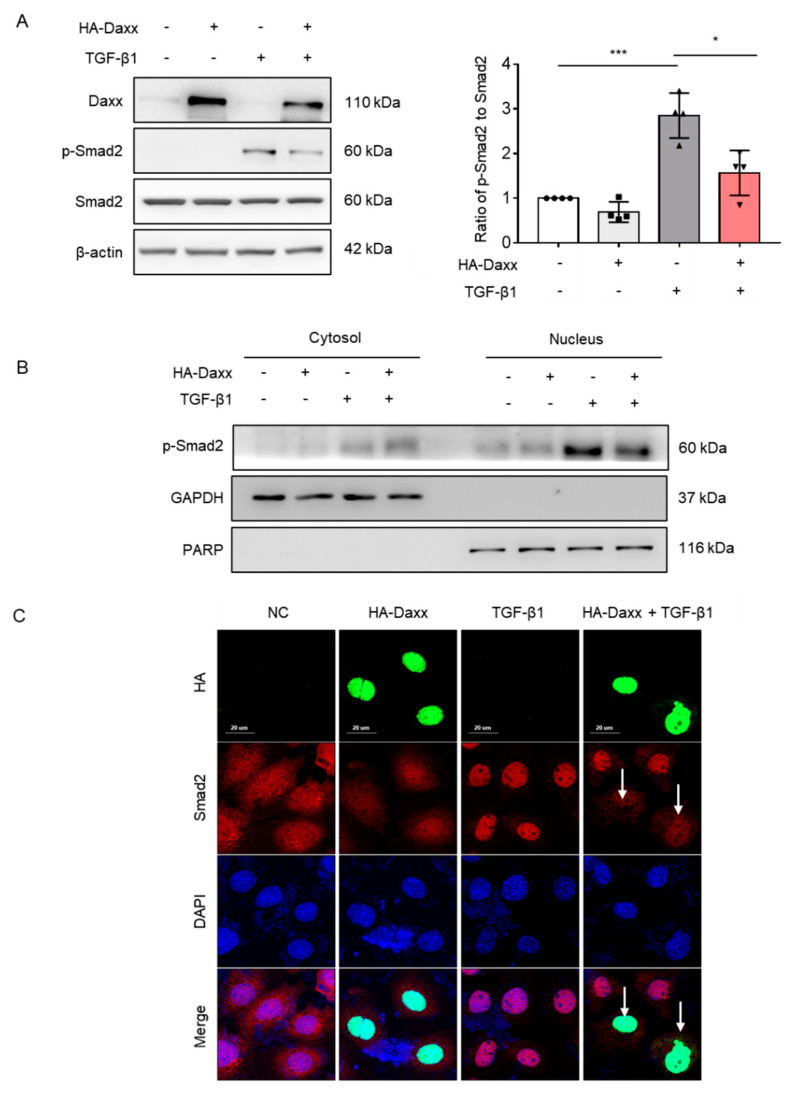
Daxx inhibited hepatocyte EMT by modulating the TGF-β signaling pathway. (**A**,**B**) FL83B cells were transfected with HA-Daxx and then treated with or without TGF-β for 24 h. (**A**) Western blotting analysis of Daxx, p-Smad2 and Smad2. The relative expression of p-Smad2 was normalized to total Smad2 expression. The graph shows quantitative densitometry from the Western blot. (**B**) Smad2 expression in the nuclear and cytoplasmic fractions of TGF-β-treated FL83B cells. (**C**) Immunofluorescence staining of Daxx (HA, green) and Smad2 (red). DAPI was used to counterstain the nucleus. Original magnification: 200×. The data are the mean ± SEM. The results are representative of at least three independent experiments. * *p* < 0.05, *** *p* < 0.001.

To determine the mechanism by which Daxx prevents TGF-β-induced hepatocyte EMT, the TGF-β signaling pathway was investigated. TGF-β activates the Smad-dependent pathway and can also affect Smad-independent pathways, including extracellular signal-regulated kinases (ERK), c-Jun N-terminal kinases (JNK), p38 MAPK and protein kinase B (AKT) pathways [22]. Thus, we assessed Smad-dependent and Smad-independent pathways by Western blotting. Interestingly, the results showed that Daxx overexpression abolished the Smad2 phosphorylation induced by TGF-β (Figure 3A), whereas the phosphorylation of other proteins, including ERK, JNK, p38 MAPK and AKT, were not affected (Figure S2). Considering that only the phosphorylated form of Smad2 can enter the nucleus and induce transcription of target genes, nuclear fractionation was performed to confirm whether Smad2 in the nucleus was regulated by Daxx. The nuclear fraction confirmed that overexpression of Daxx reduced Smad2 expression in the nucleus (Figure 3B). Then, immunofluorescence analysis was performed to confirm the intracellular locations of Smad2 and Daxx. According to the immunofluorescence analysis, Smad2 was expressed in the cytoplasm of normal cells and it entered the nucleus after TGF-β1 treatment. However, the intranuclear expression of Smad2 was decreased in Daxx-overexpressing cells specifically, even in the presence of TGF-β. (Figure 3C). These results indicate that Daxx can regulate Smad2 in the nucleus.

### 3.4. Daxx Interacts with Smad2 in the Nucleus and Regulates Transcriptional Activity

**Figure 4:**
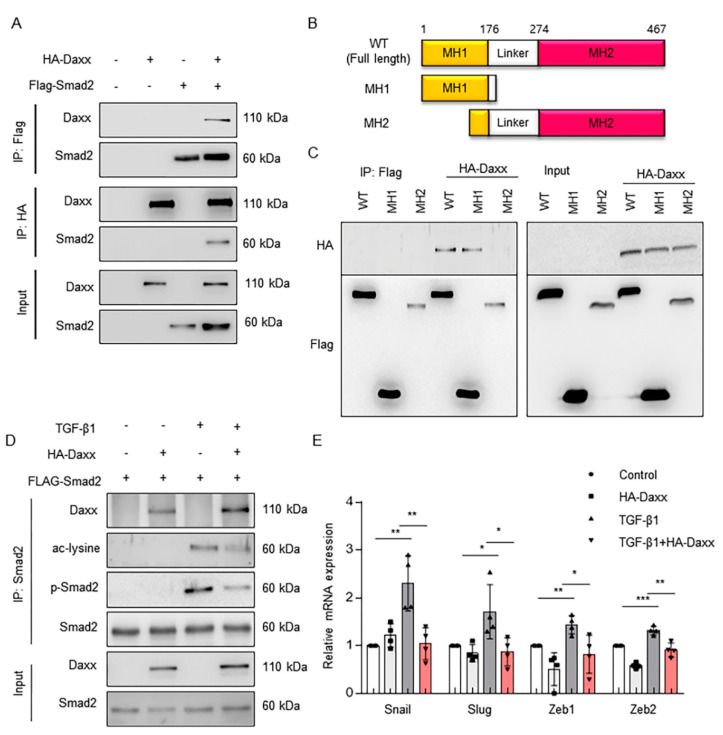
Daxx bound to the MH1 domain of Smad2 and regulated the transcription of its target genes. (**A**) HEK 293T cells were transfected with HA-Daxx and Flag-Smad2. Cell lysates were immunoprecipitated with anti-Flag beads or anti-HA antibodies. Then, precipitated proteins were separated by SDS-PAGE and detected with anti-Daxx and anti-Smad2 antibodies. (**B**) Schematic representation of full-length Smad2 (WT) and deletion mutant forms of Smad2 (each construct contains the MH1 domain or MH2 domain of Smad2). (**C**) Flag-tagged Smad2 deletion mutants were cotransfected with HA-Daxx into HEK 293T cells. Cell lysates were immunoprecipitated with anti-Flag beads. Then, precipitated proteins were detected by anti-HA and anti-Flag antibodies. (**D**) HA-Daxx- and Flag-Smad2-transfected HEK 293T cells were treated with or without TGF-β (3 ng/mL); then, the cell lysates were immunoprecipitated with anti-Smad2 antibody. The precipitated proteins were analyzed to the detect the phosphorylation and acetylation levels of Smad2. (**E**) mRNA levels of Snail, Slug, Zeb1 and Zeb2 were evaluated by qRT-PCR. The data are the mean ± SEM. The results are representative of at least three independent experiments. * *p* < 0.05, ** *p* < 0.01, *** *p* < 0.001.

Several studies have reported that Daxx functions as a transcriptional repressor of various transcription factors [17,23,24]. As Smad2 is a transcription factor, a coimmunoprecipitation assay was performed to verify whether Daxx could bind and regulate Smad2. To determine the binding ability of Daxx and Smad2, an HA-tagged Daxx plasmid and a FLAG-tagged Smad2 were cotransfected into HEK 293T cells. The results showed that Daxx can physically interact with Smad2 (Figure 4A and Figure S3A). We also found that endogenous Daxx and Smad2 binding affinity increased strongly, despite a decrease in Daxx expression after TGF-β treatment in FL83B cells (Figure S3B). Smad2 contains two conserved structural domains in the amino (MH1) and carboxyl (MH2) termini and these domains are separated by linker sequences. The MH1 and MH2 domains participate in DNA binding and transcriptional activation. Thus, we prepared MH1 (amino acids 1-185) and MH2 (amino acids 100-467), both deletion mutants of full-length Smad2, to verify which part of Smad2 could bind with Daxx (Figure 4B). The results of a coimmunoprecipitation assay using truncated Smad2 indicated that the MH1 domain of Smad2 interacted with Daxx (Figure 4C). The MH1 domain contains a major acetylation site on lysine 54 and the transcriptional activity of Smad2 in the nucleus is tightly regulated at the posttranslational level, such as via acetylation [23,24]. Thus, we detected the acetylation level of Smad2 to determine whether Smad2 acetylation was also regulated by Daxx. Our data showed that TGF-β stimulation increased Smad2 acetylation, whereas the overexpression of Daxx decreased Smad2 acetylation levels (Figure 4D). Then, we evaluated the mRNA levels of Snal1, Slug, Zeb1 and Zeb2; these are all factors that promote EMT, the transcription of which is regulated by Smad2. As expected, the mRNA levels of Snal1, Slug, Zeb1 and Zeb2 were upregulated following treatment with TGF-β1, but not in the presence of Daxx (Figure 4E). These results indicate that Daxx downregulates the transcriptional activity of Smad2 by binding to the MH1 domain of Smad2 and inhibiting its phosphorylation and acetylation.

### 3.5. Presence of Daxx Attenuates TAA-Induced Liver Fibrosis in an Animal Model

**Figure 5:**
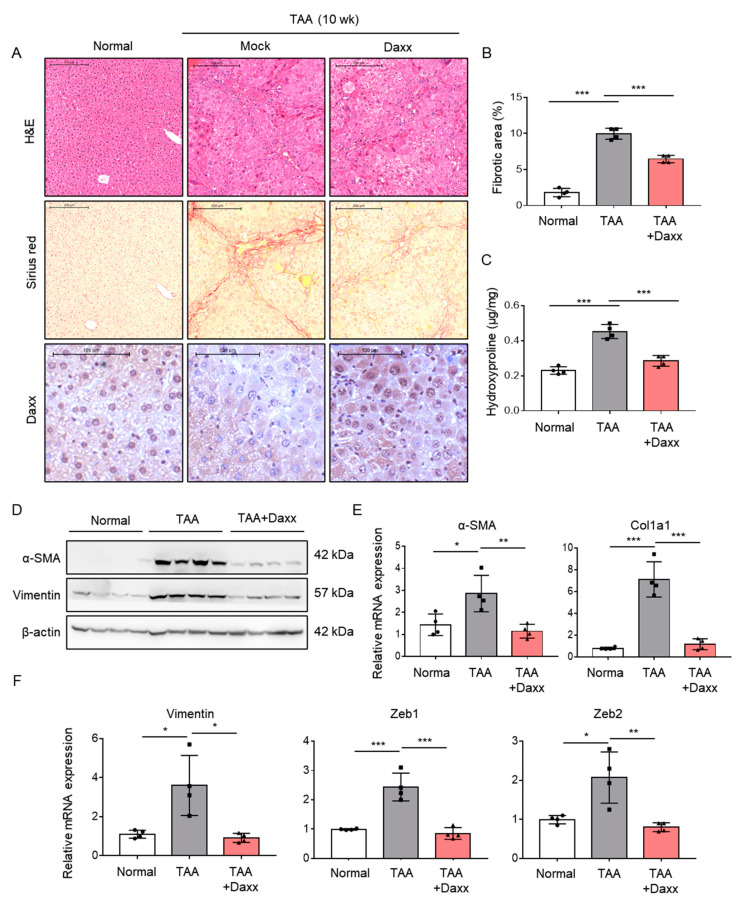
Daxx attenuated the pathological changes observed in TAA-induced liver fibrosis. (**A**–**E**) Mice were intraperitoneally injected with 100 mg/kg TAA three times a week. Hydrodynamic injection of 30 mg of Daxx plasmids was performed through the tail vein for 10 weeks (n = 4). (**A**) Morphometric analyses were performed on H&E- and Sirius red-stained liver sections. Original magnification: 50x. Dissected fibrotic liver tissues were stained with Daxx. Original magnification: 100x. (**B**) The area ratio of stained collagen was quantified from 3 different fields of 4 liver sections per group. (**C**) Hepatic hydroxyproline levels. (**D**) Western blotting analysis of α-SMA and vimentin expression; β-actin expression was used as a reference. (**E**,**F**) mRNA levels of α-SMA, Col1a1, Vimentin, Zeb1 and Zeb2 were assessed by qRT-PCR. The data are the mean ± SEM. The results are representative of at least three independent experiments. * *p* < 0.05, ** *p* < 0.01, *** *p* < 0.001.

**Figure 6:**
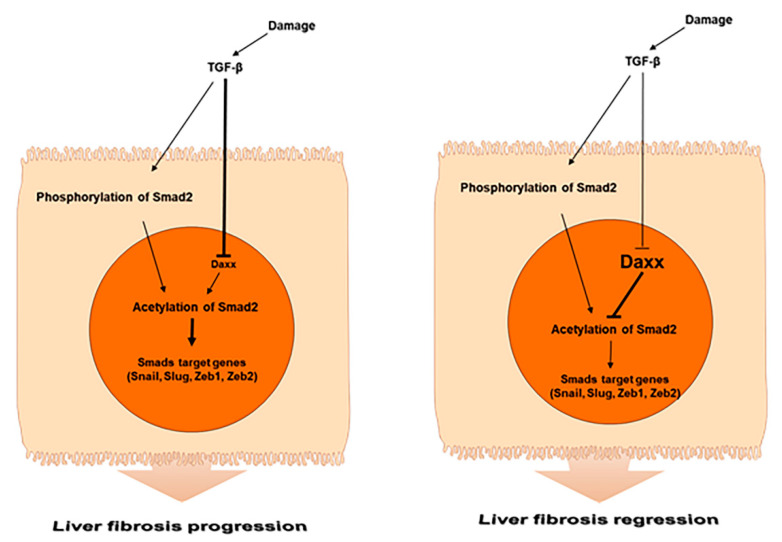
Schematic representation of the role of Daxx during liver fibrosis.

To directly evaluate the role of Daxx in liver fibrosis, we restored Daxx expression in an animal model of TAA-induced liver fibrosis. Mice injected with TAA were administered pHA-Daxx by a hydrodynamic gene delivery system. The quantification of Sirius red staining showed that the collagen deposition area was decreased by approximately 40%. The amount of hepatic hydroxyproline was significantly reduced in pHA-Daxx-treated mice compared to TAA-injected mouse livers (Figure 5A,B). In addition, Western blotting analysis indicated that the expression of α-SMA, which was upregulated after TAA administration, was suppressed when Daxx expression was restored (Figure 5C). Additionally, the mRNA levels of the fibrosis-related genes α-SMA and Collagen type 1 (Col1a1), as well as the EMT-related genes Vimentin, Zeb1 and Zeb2, were suppressed in the Daxx-injected mouse group compared to the TAA-injected mouse group (Figure 5D,E). These results demonstrate that the presence of Daxx expression ameliorates the pathological changes associated with fibrosis progression in the liver (Figure 6).

## 4. Discussion

In the present study, we determined the underlying mechanism by which Daxx functions in liver fibrosis and we identified its antifibrotic role as a key factor that modules Smad2-mediated TGF-β signaling. Specifically, we found the following: (1) Daxx expression was downregulated in human and mouse fibrotic livers. Interestingly, this downregulation was limited to the nucleus of hepatocytes of fibrotic livers. (2) Daxx inhibited the TGF-β-induced EMT process in hepatocytes. (3) Daxx interacted with the MH1 domain of Smad2 in the nucleus and regulated its transcriptional activity by repressing Smad2 protein acetylation. (4) The pathological changes in the murine fibrotic livers were improved with restored Daxx expression.

Since the first reports of EMT in hepatocytes in vitro and in vivo by Kaimori et al. [25] and Zeisberg et al. [4], many studies have shown that hepatocytes can undergo EMT and trigger liver fibrosis. There are still controversies regarding hepatocyte EMT, as one report suggested that hepatocytes do not have a collagen-expression phenotype in mice chronically treated with CCl_4_ [26]; however, in another report, the ablation of Snail, which is one of the promoting factors of EMT, in hepatocytes was shown to attenuate liver fibrosis [27]. Our data showed that the normal hepatocyte cell line FL83B acquired EMT characteristics after TGF-β treatment and that Daxx overexpression inhibited hepatocyte EMT. In addition, a fibrotic liver mouse model showed that restored Daxx expression improved pathological changes in liver fibrosis. Since HSCs are major sources of fibrogenic liver cells, it was expected that Daxx would be expressed in hepatic stellate cells; however, the IHC data indicated that Daxx was mainly expressed in hepatocytes. This suggests that, although HSCs play a pivotal role in hepatic fibrogenesis, hepatocytes, which account for approximately 80% of the liver, may also orchestrate profibrogenic responses. Thus, our findings suggest that Daxx in hepatocytes protects against the initiation or progression of liver fibrosis.

TGF-β is an important profibrogenic factor that induces the transdifferentiation of HSCs and hepatocytes to myofibroblast cells. TGF-β is a pleiotropic cytokine that is increased in various fibrotic diseases [13]. TGF-β binds to its receptor and induces the phosphorylation of Smad2/3 and phosphorylated Smad2/3 translocates to the nucleus, triggering transcription of target genes. Recent studies have revealed that acetylation of Smad2/3 plays an important role in DNA binding for the transcription of target genes. p300/CBP (histone acetyltransferase), a well-known enhancer of acetylation [28], upregulates the binding affinity of Smads to the target DNA, which results in increased transcription. Lysine 54 on the MH1 domain and lysine 378 on the MH2 domain are known to be the major acetylation sites of Smad2 that mediate TGF-β signaling [24,29]. In this study, we demonstrated that Daxx bound to the MH1 domain of Smad2 and inhibited its phosphorylation and acetylation. These findings were supported by the downregulation of Snail, Slug, Zeb1 and Zeb2, all of which are transcriptional target genes of Smad2 in Daxx-overexpressing cells. It was unclear whether the lysine 54 site binds to Daxx but, as lysine 54 is the major acetylation site of the MH1 domain and its mutation on Smad2 reduces phosphorylation and acetylation levels, the lysine 54 site of Smad2 is likely to be the direct binding site of Daxx.

Daxx has not yet been studied in liver cancer or liver fibrosis, but it has been studied in other types of cancer. Daxx acts as either a tumor suppressor or a tumor inducer by regulating several oncogenes in various types of cancer, including prostate [30], colon [31] and lung cancer [32]. In prostate cancer, Daxx promotes tumorigenicity via the suppression of autophagy [30]. In lung cancer, Daxx inhibits lung metastasis by suppressing the HIF-1α/HDAC1/Slug pathway [32]. In colon cancer, Daxx downregulation decreases E-cadherin expression via Zeb1-mediated transcriptional inhibition, leading to increased metastasis [31]. Interestingly, our results also showed that Daxx repressed the mRNA levels of Slug and Zeb1. These results are consistent with those showing that Daxx regulates the EMT-promoting factors that cause EMT-related cancer metastasis and fibrosis progression. To the best of our knowledge, this is the first study to identify Daxx as a regulator of liver fibrosis via the TGF-β signaling pathway. Based on our findings, future studies are needed to discover the association between Daxx and TGF-β signaling-induced fibrosis in other organs or liver cancer metastasis.

In summary, we demonstrated that Daxx plays a pivotal role in TGF-β-induced hepatocyte EMT by inhibiting Smad2 acetylation and phosphorylation during liver fibrosis. Accordingly, Daxx may be a potential therapeutic target for preventing and treating liver fibrosis.

## Data Availability

Not applicable.

## Supplementary Materials

The following are available online at https://www.mdpi.com/article/10.3390/cells10071742/s1, Table S1: List of primary and secondary antibodies used in Western blotting analysis, Figure S1: mRNA levels of Col1a1 and α-SMA were analyzed by qPCR, Figure S2: The expression of Daxx, p-p38, p38, p-JNK, JNK, p-ERK, ERK, p-AKT, AKT and β-actin were detected by Western blotting, Figure S3: The FL83B cells were transfected with Flag-Smad2. Cell lysates were immunoprecipitated with anti-Flag beads. Then, precipitated proteins were separated by SDS-PAGE and detected with Daxx, p-Smad2 and Smad2 antibodies.
