# Supplementary material for: Death-Associated Protein 6 (Daxx) Alleviates Liver Fibrosis by Modulating Smad2 Acetylation
Source: Cells. 2021 Jul 9;10(7):1742. doi: 10.3390/cells10071742 (PMC8305094; doi:10.3390/cells10071742)
Supplement: Supplementary file 1 [file cells-10-01742-s001.zip › cells-1256372-supplementary.pdf]

Table S1. List of primary and secondary antibodies used in western blotting analysis.

Name	Supplier	Cat no.
Daxx	Cell Signaling	#4533
α-SMA	Sigma-Aldrich	A2547
E-cadherin	Cell Signaling	#3195
Vimentin	Cell Signaling	#5741
p-Smad2	Cell Signaling	# 18338
Smad2	Cell Signaling	#5339
PARP	Cell Signaling	#9532
GAPDH	Cell Signaling	#2118
Ac-lysine	Sigma-Aldrich	06-933
p-p38	Cell Signaling	#9211
P38	Cell Signaling	#8690
p-JNK	Cell Signaling	#4668
JNK	Santa Cruz	sc-137018
p-ERK	Santa Cruz	sc-7383
ERK	Santa Cruz	sc-93
p-AKT	Cell Signaling	#4060
AKT	Cell Signaling	#9272
PCNA	abcam	ab29
β-actin	Sigma-Aldrich	A1978

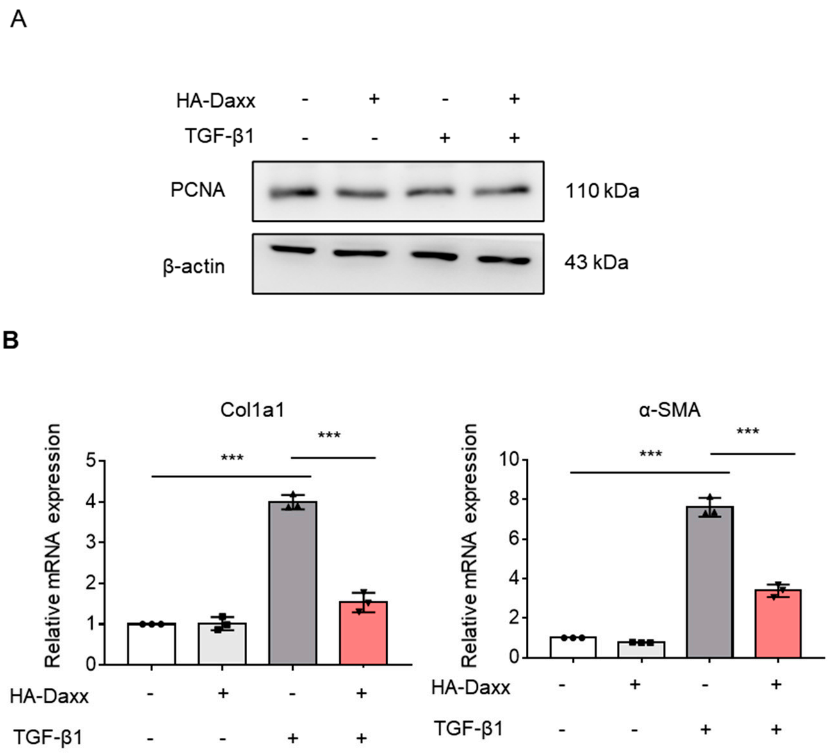

Figure S1. FL83B cells were transfected with HA-Daxx and then treated with or without TGF-β for 24 h. (**A**) Western blotting analysis of PCNA expression. Relative expression was normalized to β-actin expression as a reference. (**B**) mRNA levels of Col1a1 and α-SMA were analyzed by qPCR. All data are representative of at least three independent experiments. *** $p < 0.001$

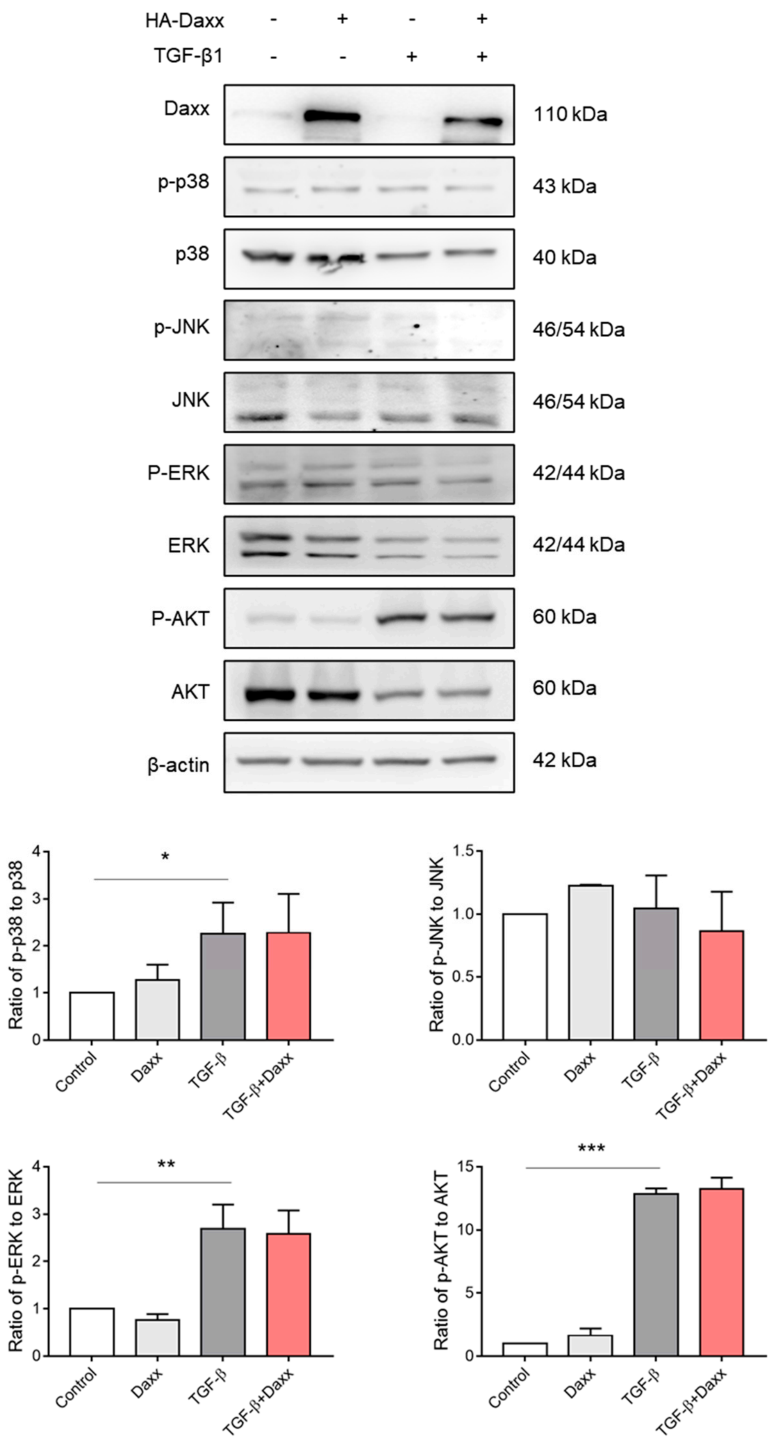

Figure S2. The expression of Daxx, p-p38, p38, p-JNK, JNK, p-ERK, ERK, p-AKT, AKT and β-actin were detected by Western blotting. The relative expression of phosphorylation was normalized to total protein level. These graphs show quantitative densitometry from the Western blot. All data are representative of at least three independent experiments. * $p < 0.05$, ** $p < 0.01$, *** $p < 0.001$.

A

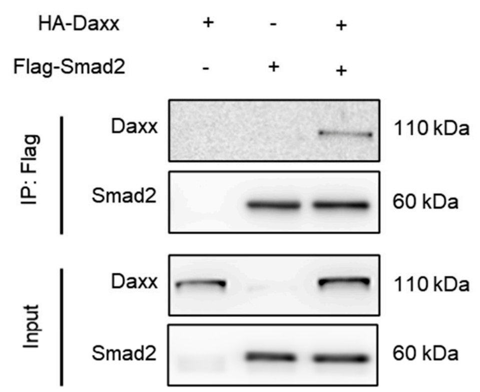

B

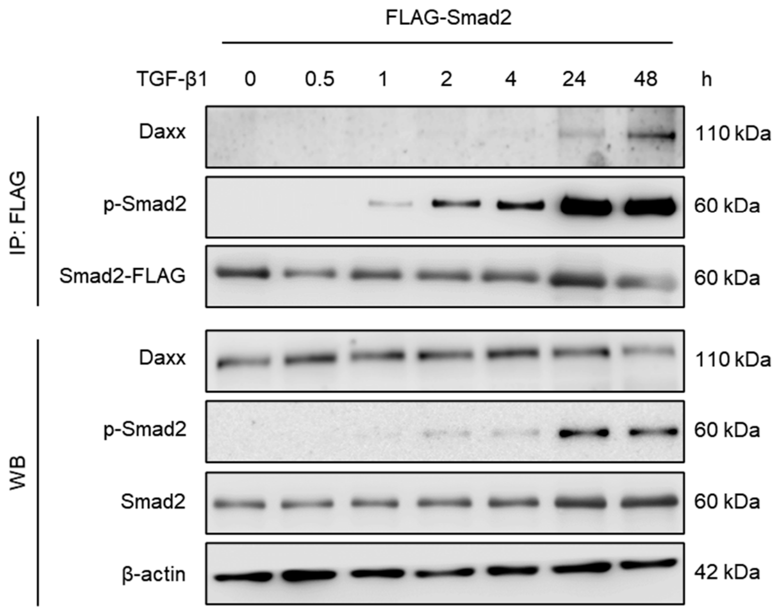

Figure S3. (**A**) HEK 293T cells were transfected with HA-Daxx and Flag-Smad2. Cell lysates were immunoprecipitated with anti-Flag beads. Then, precipitated proteins were separated by SDS-PAGE and detected with anti-Daxx and anti-Smad2 antibodies. (**B**) The FL83B cells were transfected with Flag-Smad2. Cell lysates were immunoprecipitated with anti-Flag beads. Then, precipitated proteins were separated by SDS-PAGE and detected with Daxx, p-Smad2 and Smad2 antibodies. All data are representative of at least three independent experiments.
